# Purulent pericarditis in advanced thymoma: A case report

**DOI:** 10.1016/j.radcr.2022.07.099

**Published:** 2022-08-17

**Authors:** Jordan Bakhriansyah, I Gede Parama Gandi Semita, I Gde Rurus Suryawan, Yusuf Azmi, Irfan Deny Sanjaya, Risma Ikawaty, David Nugraha, Firas Farisi Alkaff

**Affiliations:** a Faculty of Medicine, Universitas Surabaya, Jalan Raya Rungkut, Surabaya 60293, East Java, Indonesia; b Department of Cardiology and Vascular Medicine, Husada Utama Hospital, Surabaya, Indonesia; c Department of Cardiology and Vascular Medicine, Faculty of Medicine, Universitas Airlangga – Dr. Soetomo General Academic Hospital, Surabaya, Indonesia; d Department of Radiology, Faculty of Medicine Universitas Airlangga – Dr. Soetomo General Academic Hospital, Surabaya, Indonesia; e Faculty of Medicine, Universitas Airlangga, Surabaya, Indonesia; f Division of Pharmacology and Therapy, Department of Anatomy, Histology, and Pharmacology, Faculty of Medicine Universitas Airlangga, Surabaya, Indonesia; g Division of Nephrology, Department of Internal Medicine, University Medical Center Groningen, Groningen, The Netherlands

**Keywords:** Thymoma, Pericardial effusion, Mediastinal mass, Pericardiocentesis, Case report

## Abstract

Thymoma is the most common primary anterior mediastinum mass with various clinical manifestations, and one of the manifestations is pericardial effusion. While pericardial effusion in thymoma is usually serous, it can become purulent when an infection occurs in a nearby organ, albeit rare. In this report, we present a rare case of a 27-year-old woman who had purulent pericarditis secondary to an advanced thymoma. The patient came to the emergency department with the chief complaints of worsening chest discomfort, non-productive cough, and fever in the past 2 weeks. The patient was diagnosed with thymoma 5 months prior. Based on the examinations, it was discovered that the patient had pericarditis. After the pericardiocentesis was performed and the fluid was examined, the patient was diagnosed with purulent pericarditis secondary to thymoma. The patient was then treated with intravenous antibiotic and pericardial drain. Unfortunately, the patient's condition deteriorated, and the patient died on the fifth day of hospitalization. This case highlights an infrequent but potentially life-threatening complication of thymoma. In addition, thymic pathologies should be included as a rare etiology in the differential diagnosis of purulent pericardial effusion.

## Introduction

Thymoma is the most common primary anterior mediastinum mass derived from the thymic epithelium [1]. The progression of this tumor is slow and usually detected incidentally during radiographic imaging [2]. Their clinical manifestations are usually unpredictable and varies from asymptomatic to advanced symptoms such as paraneoplastic syndrome due to thoracic metastases [3]. Several symptoms related to the adjacent structure could be triggered caused by the local growth and invasion of the tumor, including pericardial effusion [3]. While the type of fluid of the pericardial effusion in this condition is usually serous, the effusion fluid can become purulent when an infection occurs in a nearby organ. In this report, we present a rare case of purulent pericarditis secondary to upper respiratory tract infection in a patient with thymoma.

## Case presentation

A 27-year-old woman presented to the emergency department with chief complaints of progressive chest discomfort, non-productive cough, and fever in the past 2 weeks. The patient was diagnosed with thymoma stage III (Masaoka-Koga) type 3B (WHO) 5 months prior and was recently underwent chemotherapy with cisplatin-etoposide regiments for 3 cycles.

On physical examination, the patient's vital signs were as follows: blood pressure 110/70 mmHg, respiratory rates 28 times / min, heart rate 145 beats per min, right axillary temperature 37.7°C, and oxygen saturation 95% at room air. No jugular venous distention was observed. The percussion on the left anterior chest was dull, and the vesicular sound was reduced. Rhonchi, wheezing, or abnormal heart sounds were absent during auscultation. The abdomen was soft and non-tender, and the liver and spleen were not palpable. Laboratory evaluation revealed elevated white blood cell (17.8 × 10^3^/μL, reference range 5-10.0 × 10^3^/μL) with neutrophils dominance (95%, reference range 55%-70%), elevated liver enzymes (aspartate transaminase190 IU/L, reference range 0-35 IU/L; alanine transaminase119 IU/L, reference range 4-36 IU/L), and elevated C-reactive protein level (14.2 mg/L, reference range <10.0 mg/L).

**Fig. 1:**
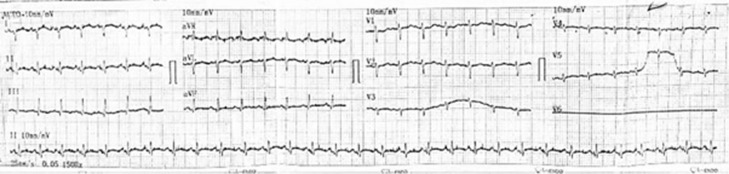
Twelve-lead electrocardiography reveals a sinus tachycardia of 150 beats per minute, right axis deviation, and a low voltage on precordial leads.

**Fig. 2:**
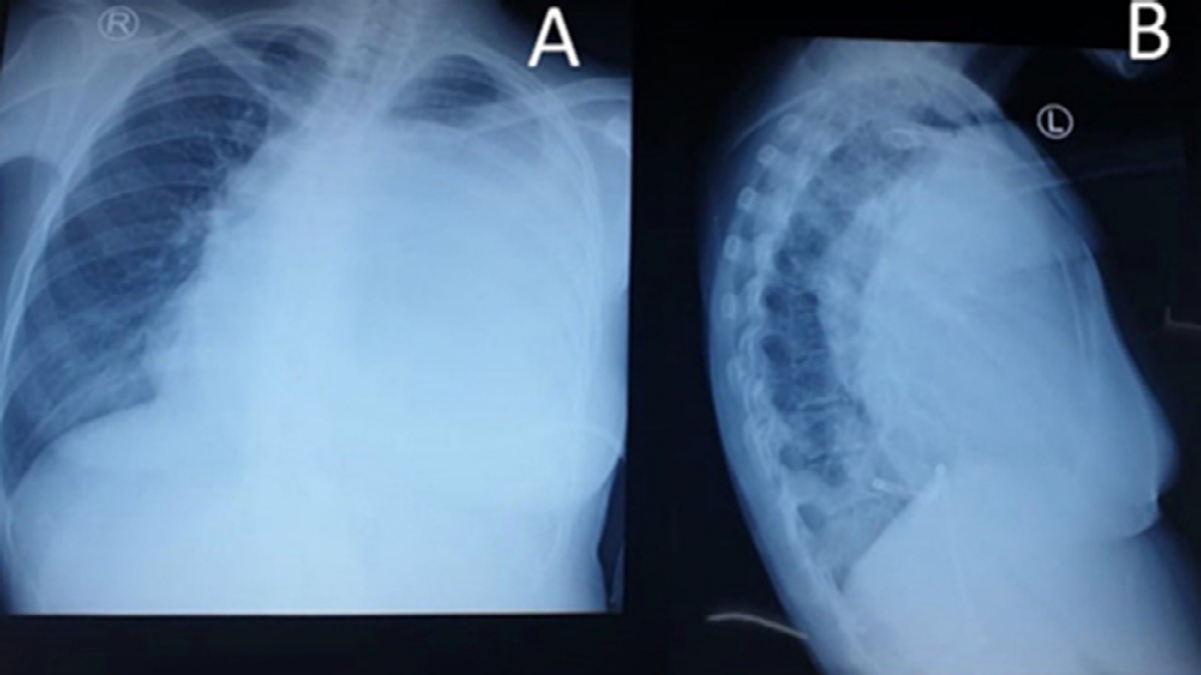
An anterior posterior (A) and lateral (B) view of chest X-ray show mediastinal widening with massive pericardial effusion.

**Fig. 3:**
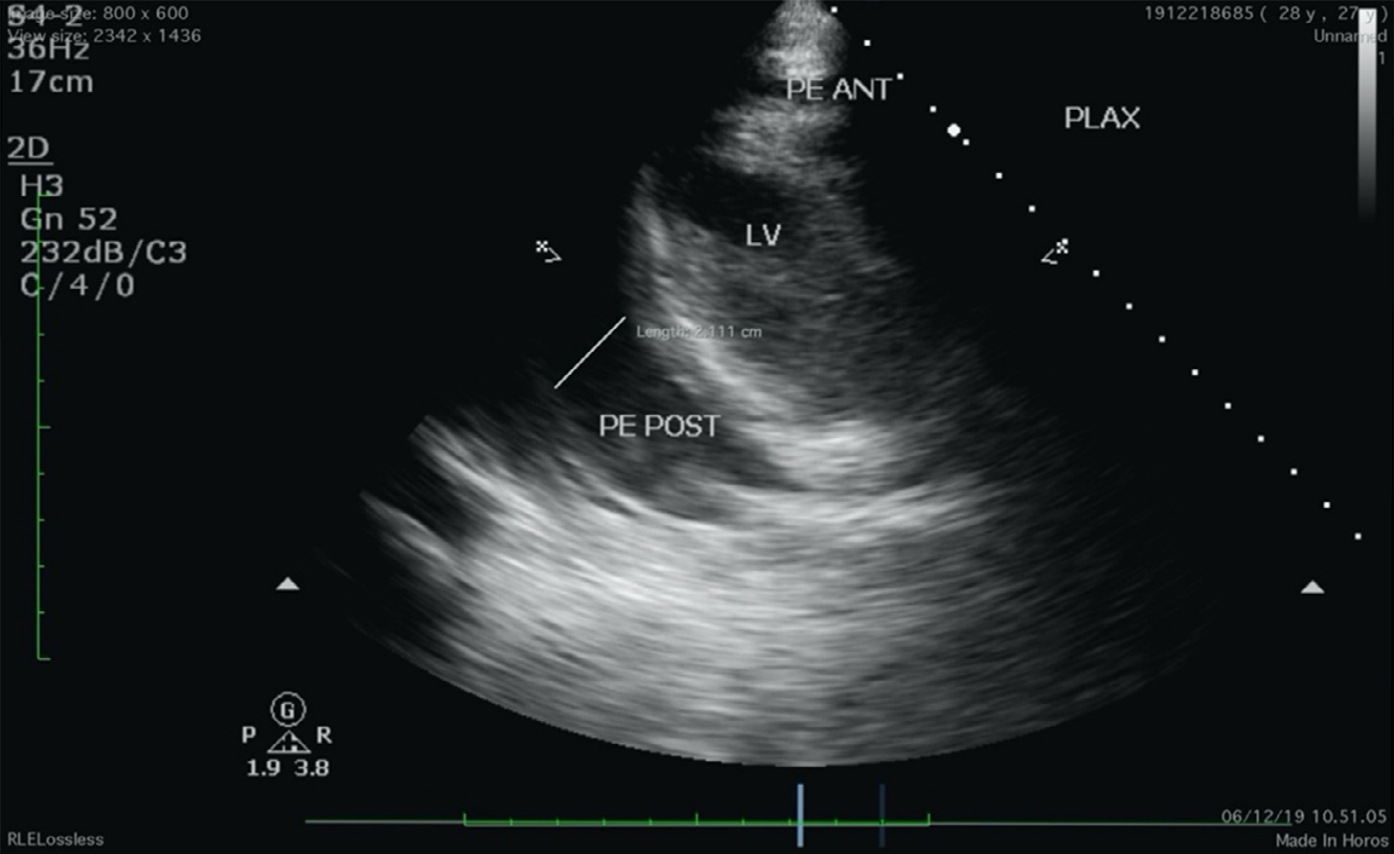
Transthoracic echocardiography demonstrates a massive pericardial effusion (21 mm) at the posterior, without any sign of right ventricle chamber collapse.

**Fig. 4:**
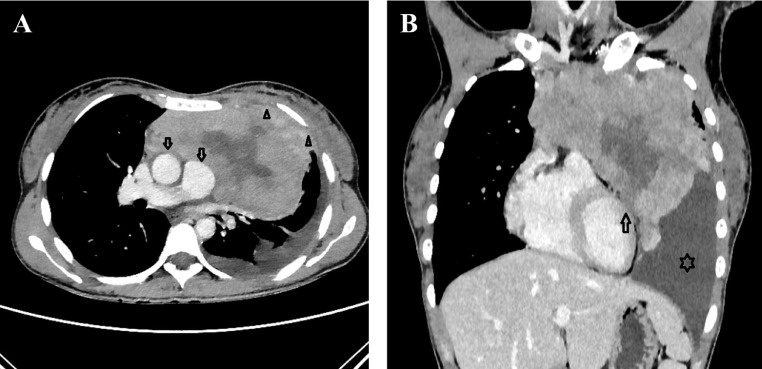
Chest computed tomography with intravenous contrast during the venous phase. (A) The axial mediastinal window shows that the anterior mediastinum contains a heterogeneously enhancing irregular mass. The mass has invaded mediastinal fat and adhered to adjacent vessels such as the aorta and pulmonary artery (arrow). The mass also adhered to the left upper hemithorax wall (open arrow). (B) The coronal mediastinal window shows the infiltration of the mass to the pericardium and epicardial fat (arrow). Also, note the presence of the left pleural effusion (asterisk).

Electrocardiography evaluation revealed sinus tachycardia, right axis deviation, and low voltage in the precordial leads (Fig. 1). Chest X-ray showed mediastinal widening with pericardial effusion and diffuse apical consolidation suggesting pneumonia (Fig. 2). Transthoracic echocardiography demonstrated a massive pericardial effusion (21 mm) at the posterior without a sign of right ventricle chamber collapse (Fig. 3). Contrast-enhanced chest computed tomography scan (CT-scan) revealed a mass sized 9.4 × 12.9 × 11.4 cm with irregular borders located at the anteromedial left mediastinum, as high as the second to fourth thoracal vertebrae. The tumor was also attached to the posterior sternum, left upper hemithorax wall, ascending aorta, aortic arch, left pulmonary artery, and left carotid artery (Fig. 4).

**Fig. 5:**
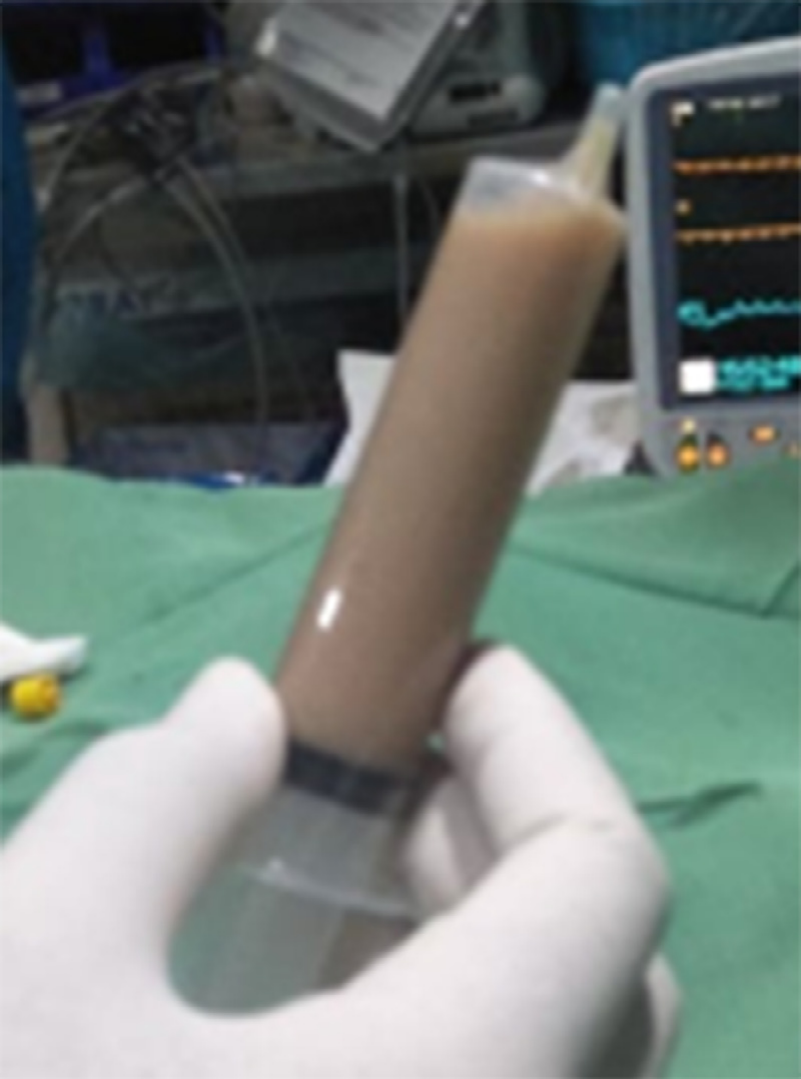
A pericardial fluid aspiration reveals thick pus in the pericardial space.

Pericardiocentesis with fluoroscopy guidance was then performed under local anesthesia, and 500 ml of thick pus was successfully aspirated (Fig. 5). The pericardial fluid analysis confirmed a purulent effusion, with elevated pericardial leucocyte count (177 × 10^3^/μL, reference range <10 × 10^3^/μL), low pericardial to serum glucose ratio (0.09, reference range > 0.3), elevated pericardial protein (4.8 g/dL, reference range > 3 g/dL), and elevated pericardial lactate dehydrogenase (963 U/L, reference range < 200 U/L). There was no malignant cells in the pericardial cytology examination. Pericardial fluid culture grew *Staphylococcus aureus*, while blood culture did not grow.

Based on the findings, the patient was diagnosed with purulent pericardial effusion secondary to stage III type B3 thymoma. The patient was treated with intravenous meropenem 1 gram thrice daily, and the pericardial drain was placed on the site of pericardiocentesis. However, 2 days afterward, the patient's condition deteriorated. The pericardial drain was not functioning properly due to loculation, and the patient was unfit for further procedures. The patient was then died on the fifth day of hospitalization.

## Discussion

Herein, we present a case of a large purulent pericardial effusion as a manifestation of an advanced thymoma. To the best of our knowledge, no prior report has been published regarding this. The incidence of thymoma is rare with only 0.15 cases per 100,000 and is commonly found in middle age and peaks in the seventh decade of life [4,5]. However, thymoma appeared at a considerably young age in our case.

The anterior mediastinum is a wholly shielded body cavity making small tumors undetected and undiagnosed. Generally, thymoma is detected when it is large enough or clinically manifests as local symptoms related to the involvement of surrounding structures, such as chest discomfort, dyspnea, hoarseness, superior vena cava syndrome. When the tumor is invasive, pericardial involvement can be found in around one-third of the cases [6,7]. Most thymoma cases are usually found through CT-scan, and this modality helps differentiate between thymoma and thymic cancer [8]. On CT-scan findings, a focal oval soft tissue mass with sharply demarcated round found in the anterior mediastinum compartment is strongly suggestive of thymoma [9]. In our cases, the CT-scan showed a heterogeneously irregular mass at the anterior mediastinum that invaded mediastinal fat and adhered to the left upper hemithorax and the adjacent vessels.

Pericardial effusion is an uncommon clinical manifestation and presents in approximately 20% of thymoma patients [10]. Pericardial effusion in our case was not caused by the primary or secondary complication of the tumor in the pericardium since the fluid cytology examination revealed no sign of malignant cell. We therefore hypothesized that the pericardial effusion was caused by the circulatory system suppression and lymphatic drainage obstruction. Lymphatic system in the heart and pericardium has a vulnerable isthmus-like section near the aortic root. The size and extent of the isthmus are not well delineated and narrow enough to cause cardiac lymphatic blockage. When there is a tumor that is located in that area, it may cause blockage of the lymphatic flow, leading to the extravasation of the lymphatic fluid to the pericardial area [11]. Next to that, when a tumor metastases to the mediastinal lymph node, it may obstruct the flow of the lymphatic drainage, causing accumulation and also extravasation of lymphatic fluid to the pericardial area [12].

Bacterial pericarditis is rarely found in the era of antibiotics, which only accounted for a less than one percent of all cases [13]. The cause of bacterial pericarditis is secondary to adjacent spreading of intrathoracic infection or by hematogenous spread [14]. In our case, we believed that the bacteria was originally from the pneumonia. Various bacteria may responsible in causing purulent pericarditis, with *Staphylococcus aureus* as the most common pathogen [15]. This is in line with the bacteria that we found in the pericardial fluid of our patient.

As the mortality rate of patients with pericarditis secondary to thymoma is high [[10], [16], [17], [18], [19]], aggressive and adequate treatments are needed. Previous studies found that patients receiving pericardiectomies had longer median survival time compared to standard supportive care and pericardiocentesis or radio-chemotherapy alone [11,20]. However, our patient had a higher mortality risk because of the advanced stage III type B3 thymoma [21]. Thus, it is not an option for our patient to undergo such procedures.

## Conclusion

In rare instances, a case of advanced thymoma can lead to more severe complications such as purulent pericardial effusion with chest discomfort and non-productive cough as the clinical manifestations. Considering the nature of this malignancy and its life-threatening prognosis, early diagnosis and prompt management of purulent pericarditis are essential.

## Patient consent

Written informed consent was obtained from the patient's husband for publication of this case report and the accompanying images.
